# How do artistic creative activities regulate our emotions? Validation of the Emotion Regulation Strategies for Artistic Creative Activities Scale (ERS-ACA)

**DOI:** 10.1371/journal.pone.0211362

**Published:** 2019-02-05

**Authors:** Daisy Fancourt, Claire Garnett, Neta Spiro, Robert West, Daniel Müllensiefen

**Affiliations:** 1 Department of Behavioural Science and Health, University College London, London, United Kingdom; 2 Centre for Performance Science, Royal College of Music, London, United Kingdom; 3 Department of Psychology, Goldsmiths, University of London, United Kingdom; University of Bologna, ITALY

## Abstract

There is a rich literature exploring emotional responses to engaging in artistic creative activities such as making music, writing, dancing and crafts. However, it remains unclear *how* such activities affect our emotions; specifically which mental processes (‘strategies’) are used to regulate our emotional responses. This paper therefore describes the design and validation of a novel instrument measuring types of emotional regulation strategies (ERSs) used when engaging in artistic creative activities: the Emotion Regulation Strategies for Artistic Creative Activities Scale (ERS-ACA). Using data from an initial pilot study (n = 740 adults, 80.4% female, median age 25–34) and a follow-up large internet sample (n = 47,924, 56.7% female, average age 47.3 ± 14.6 years), we followed a theory-driven iterative factor analysis process. Our analyses converged on a final 18-item scale comprising an overall ‘general’ factor of ERSs alongside three subscales: a 7-item factor comprising ‘avoidance strategies’ (such as distraction, suppression and detachment), a 6-item factor comprising ‘approach strategies’ (such as acceptance, reappraisal and problem solving), and a 5-item factor comprising ‘self-development strategies’ (such as enhanced self-identify, improved self-esteem and increased agency). All factors showed strong internal reliability (Cronbach’s alpha: General Factor = 0.93, Factor 1 = 0.9, Factor 2 = 0.88, Factor 3 = 0.88). We confirmed strong convergent and divergent validity, construct validity, consistency of internal reliability and test-retest reliability of the scale in a third study (n = 165, 82.2% female, average age 46.3 ± 12.2 years). In conclusion, artistic creative activities appear to affect our emotions via a number of ERSs that can be broadly classified into three categories: avoidance, approach and self-development. The ERS-ACA scale presented and validated here should support further research into the use of ERSs when engaging in artistic creative activities and enhance our understanding about how these activities affect mental health.

## Introduction

There is a rich literature of studies showing affective benefits of engaging in artistic creative activities such as making music, writing, dancing and crafts. Artistic creative activities have been shown to modulate *emotions* (rapidly-changing reactions to events in the external or internal environment), influence our *moods* (more generalised, less intense states of feeling lasting longer periods), and affect our *mental health* [1–4]. As a result, everyday artistic creative activities can impact simultaneously on immediate, mid-term and longer-term affective levels.

As emotional responses to artistic creative activities are the most immediate affective response, it is therefore important to understand *how* artistic creative activities lead to emotional engagement and processing. To facilitate this enquiry, Goethem and Sloboda proposed a four stage model [5]. First, the ‘goal’ of engaging in a creative activity refers to what somebody hopes to achieve before they start engaging in the activity (e.g. wanting to calm feelings of anxiety). Secondly, the ‘tactic’ refers to the creative activity they choose to engage in to achieve this goal (e.g. painting for an hour). Thirdly, the ‘strategy’ refers to the type of mental process used to regulate the emotions (e.g. distracting the person from their anxiety). And finally, the ‘mechanism’ mediates the induction of emotions [5] (e.g. the visual imagery conjured which occupies their thoughts). This process then leads to experiential, behavioural or physiological emotional responses to the creative activity [6].

To date, there has been significant interest in the ‘mechanisms’ (the fourth and final stage outlined above) by which certain artistic creative activities regulate our emotions. Much research on this subject has come from music psychology and has focused specifically on mechanisms such as visual imagery and rhythmic entrainment [7], and their neurological underpinnings [8]. However, our understanding of the emotion regulation ‘strategies’ (ERSs) (the third stage outlined above) underlying these mechanisms remains much less understood. Being able to identify and quantitatively capture these ERSs would allow us to understand much better what categories of strategies are used when engaging in artistic creative activities and how such strategies are affected by demographic factors (such as age, gender or training in the activity) and internal factors (such as personality or mental health). It would also allow us to understand how artistic creative activities differ from one another and thereby support the design of interventions to enhance the regulation of emotions (in the short-term), mood (in the mid-term) and mental health (in the longer-term). This is an important area of pursuit given that artistic creative activities have been shown in a number of studies to be particularly effective in regulating emotions, mood and mental health [9–16], and there is increasing use of them as adjunct therapies within health and also as recommended daily activities to support wellbeing [17–19].

Therefore, this paper describes the development and validation of a new theoretical model and novel self-report instrument designed to measure the strategies by which artistic creative activities regulate our emotions.

### Psychological literature on ERSs

Emotion regulation refers to the ability to manage one’s emotional experiences in a way that enables adaptive engagement in daily life within one’s environment. Research on emotion regulation *strategies* has grown out of two psychological traditions: psychoanalytic research (drawing on Freud’s work on anxiety regulation as a part of psychodynamic theory) [20], and stress and coping research focused on appraisal and management of demands [21]. ERSs are defined as processes that influence the intensity, duration and type of emotion experienced [22]. Gross has proposed that there are potentially limitless numbers of ERSs, but they can be differentiated along the timeline of the unfolding emotional response [23]. This model–also known as the ‘process model’–includes five different stages in the modulation of emotions that work in an interactive feedback loop. The first two stages refer to the *external situation* (such as daily hassles) and attempts to *modify* it (such as through problem solving, conflict resolution or seeking social support). The next two stages refer to the *deployment of attention*, i.e. the internal processing of the situation (such as whether one focuses on the problem or not) and *cognitive change* (such as in appraisal). The final stage involves the modulation of emotional or behavioural *response* to the previous four stages [23].

The first four of these are categorised as ‘antecedent-focused strategies’ as they involve changing either the situation, the focus on the situation or how we think about the situation. The final category is categorised as a ‘response-focused strategy’ as it involves attempts to influence the emotional response that has been elicited. Antecedent-focused strategies are also sometimes referred to as ‘cognitively-focused’ strategies and multiple examples have been identified, categorised either as ‘attentional deployment’ or ‘cognitive change’. Attentional deployment can include *concentration* on the emotion (also called ‘rumination’), where our attention is repetitively directed to our feelings or their consequences; or *distraction*, where our attention is focused away from the situation or our feelings towards. Cognitive change can include *reflection* or *reappraisal*, which involves changing a situation’s meaning in a way that alters its emotional impact; or *problem solving*, which involves specifically addressing a situation in order to try and resolve it and thereby resolve the emotions associated with it. In contrast to antecedent-focused strategies, response-focused strategies can include *suppression* of negative emotions, whereby negative emotions are squashed; or *discharge*, whereby negative emotions are released or vented [24].

However, this process model has been criticised for being too linear and ignoring the wider context of emotional experiences [25]. For example, it has been shown that stimuli can trigger emotions without cognitive reappraisal, thereby subverting the linearity of the process model [26]. So instead of this being a linear process focused on a single iterative feedback loop, it has been proposed that the external situation, internal situation and response are linked through complex bidirectional relationships [27]. Therefore, in addition to the process model, other models and categorisations have also been suggested.

One alternative categorisation that draws on the framework provided by the process model is to group strategies as ‘healthy/adaptive’ vs ‘unhealthy/maladaptive’. According to this categorisation, strategies such as reappraisal, problem solving and acceptance are adaptive while strategies such as avoidance, rumination and suppression are maladaptive [28]. Meta-analyses have shown that these ‘maladaptive’ strategies are associated with anxiety, depression and eating disorders [28]. However, there are inconsistencies, as ‘adaptive’ strategies have not been consistently found to be associated with more positive mental health [28], and reappraisal (‘adaptive’) can itself lead to rumination (‘maladaptive’) [29]. So the boundaries between ‘adaptive’ and ‘maladaptive’ may not be as clear-cut as previously assumed. This theory is supported by the fact that the successful implementation of strategies (regardless of their classification as ‘adaptive’ or ‘maladaptive’) has been consistently associated with better mental health [30].

Another categorisation that has been proposed is the dual process framework, which posits that ERSs are either ‘explicit/conscious/voluntary’ or ‘implicit/non-conscious/automatic’ [31,32]. Examples of explicit ERSs including reappraisal, distraction and suppression, while examples of implicit ERSs include adaptation and the habitualisation of certain explicit ERSs. From a neural perspective, these two categories have been shown in some studies to be regulated by different brain systems [31]. However, other neurological research has demonstrated reciprocal interactions of top-down and bottom-up processes, suggesting that these categories may be intertwined (see [25]).

A further categorisation proposed by Koole is based on targets and functions [33]. As the regulation of emotions involves manipulating a particular emotional response (such as relaxing somebody who feels stressed or energising somebody who feels despondent), Koole’s model proposes that the emotion (or emotion-generation system) that is the target of the strategy is a high-order category for classification of ERSs [33]. Three high-order categories proposed are: attention (incorporating ERSs such as distraction or suppression); knowledge (such as reappraisal), and bodily expression (such as venting or physiological self-regulation). Interacting with these targets are functions of emotion regulation. Functions can include the satisfaction of hedonic needs, the facilitation of specific goals and tasks and the optimisation of personality functioning. However, as Koole acknowledges, the categories can be permeable and occur in combination with one another [33].

Therefore, partly due to varieties in methodologies for measuring and grouping ERSs and partly due to the fluidity of many of the strategies themselves, no one model of ERSs has so far dominated. Further, there have been recent challenges to the narrowness of considerations of ERSs. One challenge has involved the potential role of mindfulness as an ERS. It has been proposed that mindfulness elicits a particular type of ERS [34]. However, it remains unclear whether mindful emotion regulation is unique from other ERSs. Mindfulness has been found to involve top-down cognitively-driven processes and bottom-up affect-driven processes and has been discussed as being relevant to all five of the stages in Gross’s process model [25]. As such mindfulness may merely be a technique cutting across a number of ERSs, although this remains to be explored further.

Another challenge has involved a call to consider the wider context of individuals and their surrounding environments in research on ERSs. Theories of embodied cognition suggest that emotion regulation happens in the context of an individual’s own adaptation, agency and relationship with the wider world around them [35]. Social cognitive theory has proposed that an individual’s self-esteem, self-efficacy and agency are intrinsically interwoven with their ability to regulate their emotions and their means of doing so [36]. However, there has been debate as to whether these aspects of ‘self’ can be argued to be an ERS in their own right. On the one hand, ERSs refer to the process of initiating, avoiding, inhibiting, maintaining or modulating the occurrence, intensity or duration of emotions, while factors such as self-esteem and self-efficacy refer to one’s perceived ability to self-regulate [36]. However, while having a high sense of self-esteem is not in itself an ERS, if an individual chooses to engage in a task that makes them feel more confident in their abilities and stronger in the face of the situation, then this perceived sense of self is arguably acting as an ERS. *Perceived sense of self* can therefore be seen as an additional ERS or category of ERSs that relates to antecedent-focused strategies in Gross’ process model (attempts to modify the situation) [23], and has been discussed alongside other ERSs such as reappraisal and acceptance as an ERS [37].

Therefore, psychological literature on ERSs attests to there being a broad range of strategies that are cognitive-, response- and self-focused, both implicit and explicit, and vary depending on the emotional target and function. While accepting that there may be limitless numbers of ERSs and personal variability in their usage [23], it is nonetheless important to find ways of categorising these ERSs in relation to specific types of activity so that we can develop our understanding of how and when they are used.

### ERSs in relation to creative activities

‘Creativity’ is complex to define, but the standard definition assumes two criteria: originality and usefulness [38]. Creativity can be considered as a ‘personality trait’, a cognitive process or a product [39]. Creativity as a personality trait includes core characteristics such as being artistic, capable, clever, curious, imaginative, individualistic, intelligent, inventive, original, resourceful and having wide interests [40]. Creativity as a cognitive process includes cognitive flexibility (such as use of multiple different perspectives), set-breaking (generating ideas in categories that are not usually thought of), and cognitive restructuring, and is further achieved through persistence and perseverance [41]. A number of studies have explored the link between these latter creative processes, moods and emotions, leading to the proposition of a dual pathway model of creativity [41,42]. This proposes that activating mood states that increase arousal support motivation and working memory capacity in a curvilinear fashion (such that low states lead to inactivity and avoidance but extremely high states lead to reduced capacity to perceive, process and evaluate information). If these mood states are positive in tone (such as feeling happy or elated), then they lead to enhanced cognitive flexibility and subsequent creative fluency and originality [43]. If they are negative in tone (such as feeling angry or stressed), then they lead to enhanced persistence and perseverance, but also with subsequent creative fluency and originality [44]. Consequently different moods can have differential effects on creativity.

In addition to research showing an emotion-creativity link, there has also been research showing the reverse: the effect of creative cognitive processes on emotions and emotion regulation. For example, in studies of children, creative cognitive processes have been associated with emotion regulation ability [45]. Creative activities that give rise to these creative cognitive processes have also been found to regulate emotions [46–48]. However, *how* different activities that promote creative cognitive processes affect the emotion regulation (i.e. the role of ERSs) remains less well understood. Consequently, this research gap formed the foundation for our study.

Given the breadth of ‘creativity’ and ‘creative processes’, we focused specifically on the *artistic* component of creativity. There have been criticisms that artistic creativity has often been given precedent within definitions of creativity and equated with creativity as a whole: a so-called ‘art bias’ [49]. Creative activities include the arts (including fine arts, crafts, literature, music and performing arts) but also other activities such as mathematics and science [50]. However, in this study, we specifically focused on artistic creativity, not to suggest a privileging of this type of creativity, but because there is growing interest in the effects of artistic creativity in relation to emotions and mental health [51–54], yet limited understanding of their effects on emotion regulation.

Within this focus on artistic creativity, there have been further distinctions between ‘Big C’ creativity (the creative genius of a selected few individuals who make remarkable discoveries and inventions) and ‘little c’ creativity (referring to creativity when engaging in everyday creative artistic activities [55]. While the distribution of Big C creative achievements is typically strongly positively skewed in samples and therefore only achieved by some, little c creativity is typically normally distributed [56]. ‘Little c’ artistic creative activities have been defined as consisting of five types: performing arts (such as singing, dancing and acting); visual arts; design and craft (such as sewing, painting and woodwork); literature (such as reading and creative writing); online, digital and electronic arts (such as photography, film-making and digital graphics design); and community and cultural festivals, fairs and events [57].In reviewing previous studies of artistic creative activities and ERSs, research undertaken to date has focused on music and ERSs, with some theoretical models proposed. In a consideration of music-induced emotions, Sakka and Juslin follow ideas of Gross’s process model in splitting strategies into categories of ‘cognitive processes’ and ‘response modulations’ [58], and also draw on ideas of Koole in incorporating physiological responses [33]. Each category then has proposed specific ERSs within it. As another alternative, Baltazar and Saarikallio also draw on Gross and Koole in referring to cognitive work and bodily reactions but their model is presented as a list of specific ERSs rather than being grouped into any overarching categories, and they mention ERSs not mentioned by Sakka and Juslin, including i) a focus on thoughts, feelings and/or situation (similar to ‘rumination’), ii) distraction, and iii) modification of feelings (similar to ‘discharge’) [59].

In studies of broader artistic creative activities, the role of specific ERSs have been discussed as part of wider measures of emotional impact. For example, a study of drawing compared venting with distraction as a means of regulating emotion through drawing [46]. And visual art has been found to improve resilience and resistance to stress [60]. But these few studies leave two challenges. First, studies that focus on just one or two ERSs arguably do not take account of the individual variation: the use of specific strategies may be driven more by factors such as personality than by a specific activity. So models that provide broader groupings of specific strategies and therefore allow some individual variation on precise choice of ERS within categories potentially provide a stronger way of conceptualising ERSs in relation to artistic creative activities. Secondly, models of ERSs that derive from studies focusing on a specific creative activity (such as music) cannot necessarily be generalised to other artistic creative activities. As many artistic creative activities are multi-modal (for acting in a play can also involve dancing to music), there is a need to develop models that will allow us to conceptualise ERSs for broad creative engagement.

### Measuring ERSs

In addition to the importance of conceptualising ERSs for artistic creative activities, it is also important to be able to measure these strategies. Only a small number of validated scales for such a purpose currently exist. One scale developed specifically to measure ERSs through engagement in music is the 40-item Music in Mood Regulation Scale [61]. This includes subscales for diversion, discharge and mental work. However, it also contains subscales that arguably are not specifically ERSs. For example, the ‘strong sensations’ subscale includes items about broad past musical experiences (e.g. ‘music has offered me magnificent experiences’), the ‘revival’ subscale includes items that could be classed instead as tactics rather than strategies (‘when I’m tired out, I rest by listening to music’), and the ‘entertainment’ subscale includes items on music listening habits (‘when I’m busy around the house and no-one else is around, I like to have some music on in the background’). Further, this scale is specific to music and so cannot be applied to other artistic creative activities.

Another scale that touches on ERSs through music engagement is the Healthy-Unhealthy Music Scale [62]. This comprises items that relate to strategies, such as a rumination item (‘I like to listen to songs over and over even though it makes me feel worse’) and a distraction item (‘Music gives me an excuse not to face up to the real world’). But it also includes items unrelated to musical strategies (e.g. ‘music leads me to do things I shouldn’t do’) and overall focuses more on the healthy vs unhealthy behavioural *outcomes* of using different ERSs. Further, the division into ‘healthy’ and ‘unhealthy’ has the same challenges discussed above [28].

Recently, Sakka and Juslin tested an emotion regulation questionnaire for listening to music [58]. This incorporated 8 items, each representing one of the following: distraction, rumination, reflection, reappraisal, acceptance, discharge, suppression and physical modulation. However, there are disputes as to whether single-item measures can adequately capture complex underlying constructs, and there are other challenges such as it not being possible to capture the internal consistency of single items, and the fact they can be more vulnerable to unknown biases in meaning and interpretation [63]. Further scales include the 21-item Music Mood-Regulation Scale (MMRS), but this measures the emotions induced (e.g. fatigue, tension, calmness, depression, etc) rather than the underlying strategies involved [64]. And the Adaptive Functions of Music Listening scale (AFML), which includes cognitive regulation, rumination and identity as subscales but also includes other scales focused more on goals than strategies (such as stress regulation, anxiety regulation, anger regulation and loneliness regulation) [65].

Outside of music psychology literature, the Self-expression and Emotion Regulation in Art Therapy Scale is a 9-item scale mainly focused around emotional discharge (e.g. ‘I am able to express my feelings through the process of making art’ and ‘I am able to depict my feelings in art therapy’) [66]. However, it also includes items unrelated to ERSs (e.g. an item on continuity of behaviour: ‘I apply the new behaviour I have been experimenting with in art therapy outside of the therapy setting’) and the wording means it cannot be applied to other artistic creative activities.

In broader psychological literature, the Emotion Regulation Questionnaire (ERQ) is a 10-item scale on a 7-point Likert scale that focuses on ERSs in daily life (rather than related to a specific activity) [6]. The scale comprises two subscales entitled ‘cognitive reappraisal’ and ‘expressive suppression’. However, the ‘cognitive reappraisal’ subscale only includes distraction and calm focus on the problem, rather than including broader cognitive strategies such as problem solving or acceptance. The scale is also narrow in scope in that it just focuses on these specific strategies rather than any others discussed above. Finally, the Cognitive Emotion Regulation Questionnaire (CERQ) has a particular focus on coping following negative situations or events and includes subscales on emotional regulation strategies such as rumination and reappraisal, but also more specific subscales on self-blame, other-blame and catastrophising [67]. But it does not cover broader response-focused strategies.

Consequently, there is a clear gap both for a new model categorising ERSs when engaging in diverse artistic creative activities and for a new validated scale for measuring these strategies. Therefore, this paper describes the development and validation of a new model and scale to measure the Emotion Regulation Strategies for Artistic Creative Activities (ERS-ACA). In Study 1, we devise an initial instrument based on previous theoretical literature and test its initial factor structure through analysing data from a pilot study of 740 adults. In Study 2, we refine the instrument and confirm its factor structure and internal reliability using data from a larger online survey of 47,924 adults. In Study 3, we assess the construct validity and retest reliability of the instrument using data from a follow-up survey of 165 adults.

## Study 1

### Materials and methods

#### Procedure

The objective of Study 1 was to devise an initial instrument and undertake the first steps in ascertaining its factor structure. The development of the scale was based on the review of the existing literature described above covering ERSs both in broad psychological literature and specific studies focused on artistic creative activities. Three researchers (DF, CG and NS) independently proposed 4–5 inventory items for each of the ERSs identified in previous studies (including acceptance, concentration, discharge, distraction, perceived sense of self, problem solving, reappraisal, reflection, rumination and suppression). Each item was worded so that it could be endorsed to varying degrees on a rating scale. The researchers were careful to ensure that statements could apply to a range of different artistic creative activities, included some negatively-worded as well as positively-worded statements and could apply to different age groups. The first version of the inventory contained 170 questions. Each item was jointly scrutinised by the researchers and items that did not adequately capture the mechanism in question, items that were ambiguous and items that were quasi-synonymous were removed. The remaining 45 items were then used in the pilot survey (see S1 Table). A 5-point response scale (strongly disagree-disagree-neither agree nor disagree-agree-strongly agree) was applied to all items.

To categorise artistic creative activities, we followed the definition proposed for population-level research [57], cross-referenced with the definition used in the UK *Taking Part* survey which collects nationally-representative data on engagement in artistic creative activities alongside broader questions on community and sports engagement [68]. This provided 16 types of participatory artistic creative activities that can be categorised as follows: performing arts (singing; dancing; playing a musical instrument; rehearsing or performing in a play/drama/opera; learning or practising magic tricks or circus skills), visual arts, design and craft (painting, drawing, printmaking, sculpture; pottery, calligraphy or jewellery making; textile crafts e.g. embroidery, crocheting or knitting; wood crafts such as carving or furniture making), literature-related (reading a novel, stories, poetry or plays for pleasure; creative writing; and composing music), and online, digital and electronic arts (creating artworks or animations on a computer; making films or videos; photography). Although there is dispute as to whether gardening and baking or cooking are ‘artistic’ activities, we also decided to include them in our study as further examples of “design or crafts” activities. We followed the theoretical standpoint that artistic creative activities are ‘multimodal’ activities [69], in that they combine multiple different components that overlap in varying degrees but that all fulfil the same basic criteria of ‘art’ proposed by Dutton [70]. For this reason, we explored ‘artistic creative activities’ together for the purposes of this study rather than as individual entities. Demographic questions on sex and age were also included.

#### Participants

The survey was publicised through the BBC’s Arts webpage and by BBC Tomorrow’s World for two weeks. This yielded responses from 978 participants. Participants were excluded if they: were under the age of 18, never undertook any of the artistic creative activities listed, provided incomplete answers to questions, or provided exactly the same response to all 45 questions (both those that were positively and negatively worded). This provided a final analytic sample size of 740. Of these, participants were 80.4% female. Just 8.0% of participants were aged 18–24, 23.7% were aged 25–34, 24.2% were aged 35–44, 23.1% were aged 45–54, 16.4% were aged 55–64, 4.3% were aged 65–74 and just 0.3% were aged over 75. For this and the following studies, all were approved by the University College London Research Ethics Committee and all participants gave informed consent. Additionally all data were analysed anonymously.

#### Statistics

Three of the 45 items showed substantial amount of skewness or kurtosis so were removed from the analysis. Four further items were judged on reflection not to adequately capture ERSs. Of the remaining 38 items, an exploratory factor analysis suggested that four factors had an eigenvalue > 1 and explained >5% each. Visual inspection of a screeplot suggested that four factors were also an acceptable solution according to Kaiser’s elbow criterion [71]. Although different minimum criteria for primary factor loadings are proposed, as this study focused on reducing the number of items in the scale, we applied a cut off of .4 or above in line with Pituch and Stevens [72]. A total of five items failed to meet a minimum criteria of having a primary factor loading of .4 or above so were excluded. A further two items showed poor discrimination, loading equally onto two different factors so were excluded. This left a total of 31 items for continuation into study 2 (see Table 1 for factor loadings and S1 Table for all items initially included in the pilot study).

**Table 1:** Items included in the ERS-ACA scale with factor loadings.

	Factor 1 (avoidance strategies)	Factor 2 (approach strategies)	Factor 3 (self-development strategies)	Factor 4 (escapism strategies)
1. …it creates a safe space for me	0.43			
2. …I am completely ‘in the zone’ and only focus on the activity itself	0.45			
3. …it helps me to come to terms with my own emotions		0.55		
4. …it redirects my attention so I forget unwanted thoughts and feelings	0.63			
5. …I am reassured of my own abilities			0.61	
6. …it helps me to think rationally about things in my life		0.62		
7. …I can block out any unwanted thoughts or feelings	0.67			
8. …it reaffirms my identity			0.53	
9. …I can contemplate what is going on in my life with a clear mind		0.67		
10. …I can shake off any anxieties in my life	0.56			
11. …it helps me to vent negative feelings such as anger		0.48		
12. …it makes me feel detached from negative things in my life	0.67			
13. …it helps me to disengage from things that are bothering me	0.69			
14. …I feel I am in my own little bubble, away from ordinary worries	0.59			
15. …it helps me to put worries or problems I have in perspective		0.68		
16. …I feel more capable of tackling challenges		0.47		
17. …I feel more confident in myself			0.75	
18. …I worry more about things in my life	0.44			
19. …it helps me to understand my own feelings on things that are on my mind		0.73		
20. …I imagine myself in another place				0.52
21. …it boosts my self-esteem			0.75	
22. …I am in my own world				0.50
23. …it gives me a sense of purpose			0.60	
24. …it helps me forget about my worries	0.68			
25. …it helps me refocus on what matter in my life		0.61		
26. …I actively plan about how I can solve worries or problems in my life		0.61		
27. …it helps me to channel feelings of sadness and misery		0.54		
28. …it makes me feel cleansed of negative feelings		0.47		
29. …It makes me feel stronger in myself			0.57	
30. …it makes me reflect on my emotions		0.70		
31. …I conjure up pleasant visual images or daydreams in my mind				0.45

### Results

Our initial exploration of the factor structure of the instrument suggested that the factors were differentiated as follows. Factor 1 comprised items that covered a range of ERSs relating to *avoidance* strategies for regulating emotions. These included detachment, distraction and suppression and detachment (e.g. “it creates a safe space for me”, “it redirects my attention so I forget unwanted thoughts and feelings”, “I can block out any unwanted thoughts or feelings” and “it helps me to disengage from things that are bothering me”).

Factor 2 comprised items that covered further ERSs relating to *approach* strategies for regulating emotions. These included acceptance, reappraisal, discharge and problem solving (e.g. “it helps me to come to terms with my own emotions”, “it helps me to think rationally about things in my life”, “it helps me to vent negative feelings such as anger”, and “I actively plan about how I can solve worries or problems in my life”).

Factor 3 comprised items that covered further ERSs relating to *improving a sense of self* including enhanced self-identity, increased self-esteem and improved agency (e.g. “it reaffirms my identity”, “I am reassured of my own abilities” and “I feel more capable of tackling challenges”).

Factor 4 comprised items that related to *escapism* including distraction and detachment (e.g. “I imagine myself in another place” and “I conjure up pleasant visual images or daydreams in my mind”.

#### Summary

Study 1 therefore involved the initial testing and refinement of the ERS-ACA scale. Our analyses proposed an initial four-factor structure comprising factors focused on avoidance strategies, approach strategies, self-development strategies and escapism strategies. This fourth factor contained just three items and showed similarities to Factor 1. However, this was a preliminary factor analysis focused on reducing items in the scale and scoping initial factor structure, so having reduced an initial 45 items to a shortened 31-item form, we sought a larger sample in order to carry out further testing in Study 2.

## Study 2

### Materials and methods

#### Procedure

The objective of Study 2 was to refine the instrument and confirm its factor structure and internal reliability. Therefore, following the pilot, the shorter 31-item scale was launched in March 2018 as part of the online *Great British Creativity Test* developed by BBC Arts and BBC Tomorrow’s World. It was promoted across the BBC broadcast network for a period of 11 weeks. During this time, 48,122 people aged 18 or over took part. Participants were excluded if they took the test more than once (n = 158) or provided exactly the same response to all ERS questions (both those that were positively and negatively worded; n = 40). This provided a final sample size of 47,924. Participants were 56.7% female, with an average age of 47.3 years (SD = 14.6, range 18–99), majority white British or Irish (90.0%) (see Table 2).

**Table 2:** Demographic details for participants in Study 2.

	N = 47,924
Sex, female %	56.7%
Age, mean (SD)	47.3 (14.6)
Ethnicity, %	
White British/Irish/Other	90.0%
Asian/Asian British/Bangladeshi/Indian/Pakistani/Other	2.7%
Black/Black British/African/Caribbean/Other	0.7%
Chinese/Chinese British	0.6%
Mixed race	1.8%
Other/prefer not to say	4.2%
Living status, %	
Living with spouse or partner	66.8%
Living with other family	9.5%
Living with friends	5.2%
Living alone	17.0%
Other	1.5%
Educational attainment, %	
No formal qualifications	2.3%
GCSE/CSE/O-levels or other age 16 attainment	7.4%
A-levels or other post-16 attainment	16.2%
Undergraduate degree	45.4%
Postgraduate degree	28.8%
Occupational status, %	
In full-time employment	46.6%
In part-time employment/self-employed	24.7%
In education	4.6%
Retired	16.7%
Not working	7.4%
Household income, %	
<£16,000	12.0%
£16,000-£29,999	20.9%
£30,000-£59,000	35.1%
£60,000-£89,000	17.4%
£90,000-£119,999	7.7%
>£120,000	6.9%
Favourite creative activity, %	
Singing	12.4%
Painting, drawing, printmaking or sculpture	12.2%
Gardening	12.0%
Reading novels, stories, poetry or play	11.9%
Playing a musical instrument	9.8%
Cookery or baking	9.8%
Textile crafts such as embroidery, crocheting or knitting	7.8%
Creative writing	6.8%
Dancing	5.5%
Photography	4.7%
Composing music	1.6%
Wood crafts such as carving or furniture making	1.6%
Creating artworks or animations on a computer	1.2%
Pottery, calligraphy or jewellery making	1.1%
Rehearsing or performing in a play/drama/opera/musical theatre	0.9%
Making films or videos	0.7%
Learning or practising magic tricks or circus skills	0.2%

#### Statistics

The full data set (N = 47,924) was randomly split into a training dataset for exploratory factor analysis (N = 23,962) and test set for confirmatory analysis (N = 23,962). As participants only submitted their responses on completing the full questionnaire and as all questions were mandatory, there were no missing data. For the exploratory factor analysis, none of the 31 variables showed any substantial amount of skewness or kurtosis (all < |2|) and hence all variables were used in the analysis. McDonald’s coefficient omega hierarchical for the training dataset was 0.76 which suggests strong evidence of a hierarchical factor structure [73]. The presence of a hierarchical factor indicates that participants in the sample differ in their overall tendency to use the arts for emotion regulation, in addition to differing with respect to individual usage strategies. Therefore, a factor model (using minimum residual factoring) with one factor was specified and the matrix of residuals from this 1-factor model was subsequently subjected to a parallel factor analysis. The parallel analysis suggested that three principal components or 11 factors would constitute solutions according to simulated data from the same variables. However, only three factors had an eigenvalue > 1 and the screeplot also suggested three factors according to Kaiser’s elbow criterion [71]. Therefore, we ran a hierarchical minimum residual factor analysis with one general factor and three group factors.

In a second step we excluded 11 items with a communality of <0.5 and subsequently two items with a substantial cross-loading on the group factors (i.e. ratio of loading on primary factor to secondary factor <2). Then, minimum residual exploratory factor analysis specifying one hierarchical and three group factors was applied to the remaining 18 items, using the function omega() from the R package psych [74]. Internally, omega() performs a factor analysis using the oblique oblimin rotation with a subsequent Schmid-Leiman transformation to identify the general factor which accounts for the correlations among group factors. In contrast to study 1, where the focus was on reducing the number of items in the scale, we applied a lower cut off of 0.32 for factor loading following Tabachnick and Fidell [75]. The resulting model had seven items with substantial loadings (i.e. group factor loadings ranged from .33 to .6) on the first factor, six items loading on the second factor and five items loading on the third factor group factor. In addition, all items showed substantial loadings (from .48 to .71) on the general factor. For the assessment of model fit we used the test data set, specified the 1+3 hierarchical factor model and computed a confirmatory factor analysis using robust maximum likelihood estimation and orthogonal rotation among group factors because the general factor accounts for the correlations among group factors. Fit indices indicated a good to very good fit of the model to the data (chi square = 4074, df = 117, p < .001; RMSEA = .053, SRMR = .026, Tucker-Lewis Index = .946, Comparative Fit Index = .959). The results from the confirmatory factor analysis are presented in Table 3.

**Table 3:** Items included in the ERS-ACA scale with factor loadings.

	General factor	Factor 1 (avoidance strategies)	Factor 2 (approach strategies)	Factor 3 (self-development strategies)
1. …I can block out any unwanted thoughts or feelings	0.52	0.56		
2. …I can shake off any anxieties in my life	0.63	0.39		
3. …I feel I am in my own little bubble, away from ordinary worries	0.54	0.45		
4. …it helps me forget about my worries	0.59	0.52		
5. …it helps me to disengage from things that are bothering me	0.58	0.60		
6. …it makes me feel detached from negative things in my life	0.57	0.51		
7. …it redirects my attention so I forget unwanted thoughts and feelings	0.48	0.54		
8. …I can contemplate what is going on in my life with a clear mind	0.63		0.39	
9. …it helps me refocus on what matter in my life	0.69		0.37	
10. …it helps me to come to terms with my own emotions	0.64		0.33	
11. …it helps me to put worries or problems I have in perspective	0.66		0.39	
12. …it helps me to understand my own feelings on things that are on my mind	0.62		0.52	
13. …it makes me reflect on my emotions	0.57		0.47	
14. …I feel more confident in myself	0.66			0.50
15. …it boosts my self-esteem	0.65			0.51
16. …it gives me a sense of purpose	0.57			0.38
17. …It makes me feel stronger in myself	0.71			0.38
18. …it reaffirms my identity	0.64			0.35

Note: all items scored from 1 (strongly disagree) to 5 (strongly agree).

### Results

Study 2 analyses suggested three factors comprising 5–7 items (see Table 3). Factor 1 comprised seven items that covered a range of ERSs including distraction, suppression and detachment (e.g. “it directs my attention so I forget unwanted thoughts and feelings”, “I can block out any unwanted thoughts or feelings” and “it helps me to disengage from things that are bothering me”). As each of these items involved an attempt to avoid or ignore negative emotions, this factor was labelled *avoidance strategies*.

Factor 2 comprised six items that covered a range of ERSs including acceptance, reappraisal and problem solving (e.g. “it helps me to come to terms with my own emotions”, “it helps me to put worries or problems I have in perspective” and “I can contemplate what is going on in my life with a clear mind”). As each of these items involved directly addressing negative emotions, this factor was labelled *approach strategies*.

Factor 3 comprised five items that covered a ERSs including enhanced self-identity, improved self-esteem and increased agency (e.g. “it reaffirms my identity”, “it makes me feel stronger in myself” and “it gives me a sense of purpose”). As each of these involved a refocusing on oneself, this factor was labelled *self-development strategies*.

All factors showed strong internal reliability (general factor, Cronbach’s alpha = 0.93, Factor 1 = 0.9, Factor 2 = 0.88, Factor 3 = 0.88). The full scale for Emotion Regulation Strategies in Artistic Creative Activities (ERS-ACA) is shown in Table 3. The normative values for factor responses by demographic variables are shown in S2 Table, and the data norms by percentile are shown in S3 Table.

### Summary

In summary, Study 2 identified a strong general factor for ERSs as well as three group factors, each representing different categories of ERSs. As anticipated from analyses in Study 1, Factor 4 was no longer present in Study 2, with none of the items loading sufficiently onto the other factors. These other factors showed stability and consistency with the findings from Study 1, lending confidence to our findings. This study also enabled the refinement of the scale to just 18 items, with this instrument showing strong internal reliability.

## Study 3

### Materials and methods

#### Procedure

The objective of Study 3 was to test the convergent and divergent validity of the instrument (its correspondence with measures of a similar construct and its distinctness from measures of other constructs [76]), confirm the consistency of its internal reliability over time and ascertain its test-retest validity. Therefore, a new survey containing the 18-item ERS-ACA scale was publicised through social media for one week. This yielded complete responses from 165 participants, which were used for analyses of convergent and divergent validity and correlation with related constructs. Of these, 119 participants then repeated the survey 2 weeks later, with their responses used for analyses of test-retest reliability. Participants in the baseline were 82.2% female. Participants had an average age of 46.3 years (SD = 12.2, range 23–71). In addition to answering the 18-item ERS-ACA scale, participants also responded to the subscales for ‘diversion’, ‘discharge’ and ‘mental work’ from the 21-item Brief Music in Mood Regulation Scale [61]; and the 13-item Healthy-Unhealthy Music Scale [62]. For the Brief Music in Mood Regulation Scale, we specifically only used these subscales as we did not consider the other subscales (entertainment, revival, strong sensations and solace) to be focusing specifically on ERSs but more on *tactics* (for ‘entertainment’ and ‘revival’) and broader experiences (for ‘strong sensations’). For both the Health-Unhealthy Music scale and the Brief Music in Mood Regulation Scale, the word ‘music’ was modified to ‘creative activity’. We also compared the performance of ERS-ACA with the 10-item Emotion Regulation Questionnaire (ERQ) which measures general emotion regulation strategies in daily life in order to identify whether ERS-ACA merely captured trait ERSs rather than state ERSs when engaging in creative activities [6].

#### Statistics

Pearson’s r correlation coefficients were used to explore the relationship between our ERS-ACA scale and the other related ERSs scales. Cronbach’s alphas were computed for the scale at both time points to confirm the stability of internal consistency of the scale. For test-retest validity, correlations of participants’ test scores at baseline and two-week follow-up were then computed using Pearson’s r and Spearman’s rho correlation coefficients, and the individual intra-class correlation coefficient (ICC) with a 2-way random model with absolute agreement was calculated [77].

### Results

*Convergent and divergent validity*: Factor 1 (avoidance strategies) demonstrated convergent validity with the ‘diversion’ subscale of the Brief Music in Mood Regulation Scale (B-MMRS), showing a large positive correlation (see Table 4). Factor 1 also showed divergent validity with ‘discharge’ and ‘mental work’ subscales, with no significant associations present. Factor 2 (approach strategies) showed convergent validity with ‘discharge’ and in particular ‘mental work’ (for which there was a strong positive correlation). Factor 2 also showed a small-medium correlation with diversion which was unexpected. Factor 3 showed medium correlations with all subscales of B-MMRS.

**Table 4:** Pearson correlations from convergent and divergent validity tests.

	Factor 1: Avoidance strategies	Factor 2: Approach strategies	Factor 3: Self-development strategies
Brief Music in Mood Regulation Scale			
Diversion	r = 0.56 p < .001	r = 0.36 p < .001	r = 0.41 p < .001
Discharge	r = 0.13 p = .097	r = 0.42 p < .001	r = 0.31 p < .001
Mental work	r = 0.12 p = .13	r = 0.71 p < .001	r = 0.49 p < .001
Healthy-Unhealthy Music Scale			
Healthy subscale	r = 0.48 p < .001	r = 0.46 p < .001	r = 0.68 p < .001
Unhealthy subscale	r = -0.20 p = .011	r = 0.02 p = .76	r = -0.15 p = .066
Emotion Regulation Questionnaire			
Cognitive reappraisal	r = 0.04 p = .60	r = 0.27 p < .001	r = 0.24 p = .003
Expressive suppression	r = -0.16 p = .058	r = -0.15 p = .061	r = -0.27 p = .001

We also examined the correlation of ERS-ACA with the Healthy-Unhealthy Music Scale which touches on ERSs but in fact focuses on ‘healthy’ vs ‘unhealthy’ behavioural outcomes from the use of ERSs. All three ERS-ACA factors were positively associated with the ‘healthy’ subscale of the Health-Unhealthy Music Scale. However, neither Factor 2 nor Factor 3 (self-development strategies) were associated with the unhealthy subscale, and Factor 1 (avoidance) showed a weak inverse correlation with the unhealthy subscale (see Table 4).

Finally, we compared ERS-ACA with the Emotion Regulation Questionnaire, which measures an individual’s tendency to regulate their emotions in a particular way more broadly in their lives. There were only small associations between having a broad tendency to use ‘cognitive reappraisal’ in daily life with the use of Factors 2 and 3 when engaging in artistic creative activities. Having a broad tendency to use ‘expressive suppression’ was not associated with either Factors 1 or 2 but was negatively correlated with to a small degree with Factor 3.

*Reliability*: We tested the consistency of the internal reliability assessed in Study 2. At baseline, Cronbach’s alphas for the whole scale and three factors were 0.90, 0.87, 0.89 and 0.86 respectively. At follow-up, they were consistently strong: 0.92, 0.90, 0.86 and 0.90 respectively. We also tested the test-retest validity of the scale (see Table 5). Pearson’s correlation coefficient showed a strong correlation ERS-ACA ratings at first measurement and the measurement two weeks later (r = 0.85, p < .001), and this was confirmed by Spearman’s correlation coefficient (rho = 0.80, p = .001). Our intra-class correlation coefficient for individual ratings for both the general factor and Factors 2 and 3 ‘good’ (indicated by ICC 0.75–0.9), while Factor 1 was ‘moderate’ (ICC 0.5–0.75) [77]. These results all confirmed strong reliability properties of the scale.

**Table 5:** Test-retest validity of the ERS-ACA scale.

	Pearson’s correlation	Spearman’s correlation	Individual intraclass correlation coefficient (ICC)	95% Confidence Interval (CI)
General factor	0.85, p < .001	0.80, p < .001	0.83	0.74–0.89
Factor 1 (avoidance strategies)	0.72, p < .001	0.69, p < .001	0.71	0.60–0.80
Factor 2 (approach strategies)	0.80, p < .001	0.73, p < .001	0.80	0.72–0.86
Factor 3 (self-development strategies)	0.82, p < .001	0.75, p < .001	0.81	0.73–0.87

### Summary

Overall, the scale showed good convergent validity. Avoidance strategies (Factor 1) were strongly associated with the ‘diversion’ subscale of the Brief Music in Mood Regulation Scale (B-MMRS) but not associated with ‘discharge or ‘mental work’. This was as expected, given that ‘diversion’ is an ERS that involves avoidance, while ‘discharge’ and ‘mental work’ both involve addressing problems. This suggested that this factor performed exactly as expected in relation to the B-MMRS scale. Factor 2 (approach strategies) showed convergent validity with ‘discharge’ and ‘mental work’, which are both approach-focused. However, we found an unexpected correlation between approach strategies and the ‘diversion’ subscale. The explanation for this could lie in challenges with the B-MMRS scale itself. The scale validation of B-MMRS found that diversion and mental work showed large cross-loadings, suggesting a lack of discrimination within B-MMRS between these constructs. But it is overall promising that the ERS-ACA factors aligned with the relevant subscales of B-MMRS. Self-development strategies (Factor 3) correlated with all three factors from the B-MMRS. This could suggest that Factor 3 does not possess as strong convergent or divergent properties. However, we made no a priori hypotheses for the performance of Factor 3 as much less research has been undertaken on self-development strategies, as outlined in our literature review. As such, there was no existing scale that we felt was adequate to test the convergent or divergent validity of Factor 3.

Our construct validity tests with the Healthy-Unhealthy Music Scale confirmed approach and self-development strategies as correlating with the ‘Healthy’ subscale, which was as expected given previous literature identifying cognitively-focused strategies as ‘adaptive’ [78]. These results are discussed further in the general discussion below.

We also tested whether ERS-ACA was merely measuring trait ERS through using the Emotion Regulation Questionnaire. However, we found only weak associations with trait ERSs, suggesting that when completing ERS-ACA in relation to their responses to creative activities, individuals do not merely report broader emotion regulation strategies in daily life. Finally, the scale also showed comparably strong alphas in its testing in Study 3 as well as good intraclass correlation over a two-week test-retest period.

In summary, Study 3 confirmed that the ERS-ACA scale performs as expected against existing measures, exhibiting good convergent and divergent validity. It also shows strong performance in tests of internal reliability and test-retest validity.

## General discussion

This paper has reported the development and validation of a novel instrument to measure ERSs when engaging in artistic creative activities. Drawing on a pilot data set (n = 740), a large data sample (n = 47,924) and a re-testing data set (n = 165), the construct was found to be best described by three different factors in addition to one general factor. The scale possesses high internal reliability and consistency of reliability, good convergent and divergent validity, clear construct validity, and strong test-retest reliability. The overall scale along with instructions for use and scoring is presented in S1 File.

The development of this scale was started from the Grossian standpoint that there are potentially limitless numbers of ERSs [23]. However, how these strategies group when undertaking artistic creative activities was considered. The analysis suggests three main factors. The first two of these can be categorised as ‘avoidance strategies’ (such as distraction, suppression and detachment) and ‘approach strategies’ (such as acceptance, reappraisal and problem solving). These two factors do not align with the ‘antecedent/cognitive vs response’ focused groupings proposed in Gross’ process model [23]. In Gross’ model, distraction and reappraisal are both grouped as ‘antecedent’ yet they loaded onto separate factors in our analysis. They also do not align with the ‘explicit vs implicit’ model [31,32], as suppression and reappraisal are both ‘explicit’ yet loaded separately in our analyses. However, our factor structure does have construct similarities to the ‘adaptive vs maladaptive’ model [78]. A clear distinction can be seen between the two first factors in distinguishing between those that avoid compared with those that address. It is likely that different neural networks of emotion regulation underlie the distinction that was found through the factor analysis. Indeed, Dörfel et al. have found that detachment, distraction and suppression (which all were included in the category of ‘avoidance strategies’) increase brain activation in the same regions of the right fronto-parietal network, reducing activation of the left amygdala, while reinterpretation (which was included in the category of ‘approach strategies’) involves different neural networks [79]. However, despite the construct similarities, the use of these terms ‘adaptive’ and ‘maladaptive’ is not proposed here for two reasons. First, literature discussed earlier suggests that their effects are not so clearly distinguishable as better or worse for health [78]. Further, as part of our convergent validity testing, we compared our subscales with the subscales of the Healthy-Unhealthy Music Scale. This scale touches on ERSs but in fact focuses on behavioural outcomes from the use of ERSs (for example ‘I feel happier after playing or listening to music’ and ‘Music leads me to do thing I shouldn’t do’). Our analyses showed that all three ERS-ACA subscales correlated with medium-large effect sizes with the ‘healthy’ subscale. This suggests that whatever ERSs are being employed (whether avoidance, approach or self-development), they are linked with positive ‘healthy’ behavioural outcomes, supporting previous research showing that the successful implementation of strategies (regardless of what they are) is associated with better mental health [30]. Further, it is notable that the only subscale of ERS-ACA to correlate with the ‘unhealthy’ subscale of the Healthy-Unhealthy Music Scale is the ‘avoidance’ subscale, which could be seen as ‘maladaptive’ but in fact has a negative correlation with ‘unhealthy’. This confirms that even for ‘avoidance’ behaviours when taking part in artistic creative activities, there are no ‘unhealthy’ outcomes from the use of these ERSs.

The third factor specifically focused on self-development, including self-identity, self-esteem and agency. This factor expands on many common models of ERSs by considering emotion regulation as something that happens in the context of an individual’s own adaptation, agency and relationship with the wider world around them [35]. Drawing on theories of embodied cognition, this approach suggests that perceiving oneself as strong, confident and able increases one’s abilities to regulate their emotions. Interestingly, our correlation analyses with the Emotion Regulation Questionnaire about broad daily habits showed people who made greater use of this ‘self-development strategies’ when engaging in artistic creative activities were more likely to use cognitive reappraisal strategies in their daily lives (such as rationalising stressful situations and trying to rethink their approach to negative events) and less likely to suppress their emotions (such as by keeping emotions to themselves).

This study had a number of strengths, including its multi-stage development process including initial, exploratory and confirmatory factor analysis, its multiple testing of reliability (including convergent and divergent validity, construct validity, internal reliability and test-retest validity), and its use of large sample sizes (totalling 48,829 participants overall). However, it also had some limitations. First, in using this scale, it is important to note that our development approach was theory-driven. A contrasting approach that was less theory driven could have yielded a different model, but a theory-driven approach was felt to be most appropriate as it drew on existing literature and proposed strategies that have been tested in previous studies. Further, in undertaking this study, we assessed whether specific artistic creative activities can lead to the engagement of specific ERSs. We did not specify a situation that participants needed to consider nor did we experimentally manipulate emotions. Consequently, while we have been able to validate a new measure of ERSs in relation to artistic creative activities, it remains unknown how people employ these specific ERSs in the context of different emotional situations. Research has suggested that the type of situation can dictate the type of ERS employed (for example low-intensity stimuli leading to a tendency to reappraise but high-intensity stimuli leading to a tendency to distract) [80], so this remains an important research question for future studies. Relatedly, this study involved a general healthy sample rather than a clinical sample. Further research using this measure in studies of particular participant groups such as people with diagnosed mental health conditions would help to develop our understanding of emotion regulation through creative activities. Additionally, the samples in this study were predominantly White British. It would be interesting to test this scale in broader samples to test its wider contextual applicability. The gender balance also varied across the studies, so future studies may want to consider more closely the differential emotional response of men and women to creative activities. Finally, in this study we focused specifically on validating the scale for use in relation to artistic creative activities broadly. Future studies might also like to use this scale to explore further the potential differences in ERSs when the same individuals engage in different artistic creative activities.

In conclusion, artistic creative activities appear to affect our emotions via a number of ERSs that can be broadly classified into three categories: avoidance, approach and self-development. Emotion regulation is increasingly considered a central component of mental health given that it drives individuals’ abilities to manage their emotional experiences and adapt to daily life, and has been shown to influences a number of mental health conditions [81]. The development and validation of the ERS-ACA scale should support further research into the use of these ERSs when engaging in artistic creative activities and enhance our understanding about how these activities affect mental health.

## Supporting information

S1 Table Questions included in Study 1 and 2. (DOCX)

S2 Table Normative values for factor responses (means and standard deviations). (DOCX)

S3 Table Data norms for all factors. (DOCX)

S1 File The ERSCA scale. (DOCX)
