# Supplementary material for: How do artistic creative activities regulate our emotions? Validation of the Emotion Regulation Strategies for Artistic Creative Activities Scale (ERS-ACA)
Source: PLoS One. 2019 Feb 5;14(2):e0211362. doi: 10.1371/journal.pone.0211362 (PMC6363280; doi:10.1371/journal.pone.0211362)
Supplement: S1 Table — (DOCX) [file pone.0211362.s001.docx]

**S1 Table: Questions included in Study 1 and 2**

|  | Included in Study 1 | Included in Study 2 |
| --- | --- | --- |
| …it acts as a stress relief | X |  |
| …it creates a safe space for me | X | X |
| …even though I may be doing the activity on my own, I don’t feel alone | X |  |
| …it makes me feel more stressed | X |  |
| …I accept myself for who I am | X |  |
| …I am completely ‘in the zone’ and only focus on the activity itself | X | X |
| …it helps me to come to terms with my own emotions | X | X |
| …I am proud to be doing the activity | X |  |
| …it redirects my attention so I forget unwanted thoughts and feelings | X | X |
| …I am reassured of my own abilities | X | X |
| …it helps me to think rationally about things in my life | X | X |
| …I can block out any unwanted thoughts or feelings | X | X |
| …it reaffirms my identity | X | X |
| …I can contemplate what is going on in my life with a clear mind | X | X |
| …I can shake off any anxieties in my life | X | X |
| …I feel less confident in myself and my abilities – | X |  |
| …it helps me to vent negative feelings such as anger | X | X |
| …it makes me feel detached from negative things in my life | X | X |
| …I feel I am experiencing the present moment fully | X |  |
| …it helps me to disengage from things that are bothering me | X | X |
| …I feel I am in my own little bubble, away from ordinary worries | X | X |
| …I find the familiarity of the activity reassuring and comforting | X |  |
| …it helps me to put worries or problems I have in perspective | X | X |
| …I feel more capable of tackling challenges | X | X |
| …I feel more confident in myself | X | X |
| …I feel physically aware of my own body | X |  |
| …I worry more about things in my life | X | X |
| …I find solace from things that are troubling me | X |  |
| …I get into the ‘flow’ of doing the activity | X |  |
| …it helps me to understand my own feelings on things that are on my mind | X | X |
| …I imagine myself in another place | X | X |
| …it boosts my self-esteem | X | X |
| …it calms my mind | X |  |
| …it creates a nice atmosphere | X |  |
| …I am in my own world | X | X |
| …it gives me a sense of purpose | X | X |
| …it gives me the space to focus on myself | X |  |
| …it helps me forget about my worries | X | X |
| …it helps me refocus on what matter in my life | X | X |
| …I actively plan about how I can solve worries or problems in my life | X | X |
| …it helps me to channel feelings of sadness and misery | X | X |
| …it makes me feel cleansed of negative feelings | X | X |
| …It makes me feel stronger in myself | X | X |
| …it makes me reflect on my emotions | X | X |
| …I conjure up pleasant visual images or daydreams in my mind | X | X |
