# Supplementary material for: How do artistic creative activities regulate our emotions? Validation of the Emotion Regulation Strategies for Artistic Creative Activities Scale (ERS-ACA)
Source: PLoS One. 2019 Feb 5;14(2):e0211362. doi: 10.1371/journal.pone.0211362 (PMC6363280; doi:10.1371/journal.pone.0211362)
Supplement: S2 Table — (DOCX) [file pone.0211362.s002.docx]

**S2 Table: Normative values for factor responses (means and standard deviations)**

|  | General Factor | Factor 1 (avoidance strategies) | Factor 2  (approach strategies) | Factor 3  (self-development strategies) |
| --- | --- | --- | --- | --- |
| Sex |  |  |  |  |
| Female | 3.39 (0.71) | 3.52 (0.80) | 3.15 (0.83) | 3.52 (0.85) |
| Male | 3.67 (0.64 | 3.82 (0.71) | 3.41 (0.78) | 3.76 (0.78) |
| Age |  |  |  |  |
| 18-34 | 3.56 (0.68) | 3.69 (0.77) | 3.32 (0.83) | 3.65 (0.83) |
| 35-49 | 3.54 (0.68) | 3.69 (0.76) | 3.29 (0.80) | 3.63 (0.83) |
| 50-64 | 3.55 (0.69) | 3.70 (0.77) | 3.29 (0.81) | 3.67 (0.82) |
| 65+ | 3.55 (0.71) | 3.67 (0.78) | 3.27 (0.81) | 3.71 (0.81) |
| Ethnicity |  |  |  |  |
| White British/Irish/Other | 3.55 (0.68) | 3.69 (0.76) | 3.29 (0.80) | 3.65 (0.82) |
| Other/prefer not to say | 3.57 (0.74) | 3.68 (0.81) | 3.36 (0.87) | 3.66 (0.88) |
| Living status, % |  |  |  |  |
| Living with other people | 3.54 (0.68) | 3.69 (0.76) | 3.29 (0.81) | 3.65 (0.82) |
| Living alone | 3.57 (0.70) | 3.70 (0.78) | 3.32 (0.82) | 3.68 (0.84) |
| Educational attainment, % |  |  |  |  |
| Any qualifications up to GCSE/CSE/O-levels or other age 16 attainment | 3.51 (0.74) | 3.69 (0.82) | 3.25 (0.83) | 3.58 (0.85) |
| A-levels or other post-16 attainment | 3.53 (0.70) | 3.69 (0.79) | 3.27 (0.83) | 3.62 (0.83) |
| Undergraduate degree | 3.55 (0.67) | 3.69 (0.76) | 3.28 (0.80) | 3.66 (0.81) |
| Postgraduate degree | 3.57 (0.67) | 3.69 (0.76) | 3.34 (0.81) | 3.69 (0.82) |
| Occupational status, % |  |  |  |  |
| In full-time employment | 3.49 (0.69) | 3.64 (0.77) | 3.23 (0.81) | 3.56 (0.83) |
| In part-time employment/self-employed | 3.63 (0.67) | 3.75 (0.75) | 3.38 (0.80) | 3.77 (0.80) |
| In education | 3.56 (0.69) | 3.69 (0.77) | 3.28 (0.79) | 3.70 (0.79) |
| Retired | 3.61 (0.69 | 3.70 (0.79) | 3.43 (0.85) | 3.69 (0.84) |
| Not working | 3.59 (0.70) | 3.74 (0.79) | 3.33 (0.82) | 3.68 (0.84) |
| Household income, % |  |  |  |  |
| <£16,000 | 3.64 (0.69) | 3.75 (0.79) | 3.40 (0.82) | 3.76 (0.82) |
| £16,000-£29,999 | 3.60 (0.67) | 3.72 (0.76) | 3.35 (0.80) | 3.72 (0.81) |
| £30,000-£59,000 | 3.55 (0.67) | 3.70 (0.76) | 3.30 (0.80) | 3.66 (0.81) |
| £60,000-£89,000 | 3.51 (0.67) | 3.66 (0.76) | 3.24 (0.80) | 3.60 (0.82) |
| £90,000-£119,999 | 3.46 (0.68) | 3.62 (0.75) | 3.22 (0.82) | 3.54 (0.82) |
| >£120,000 | 3.42 (0.72) | 3.58 (0.79) | 3.17 (0.83) | 3.49 (0.87) |
| Favourite creative activity, % |  |  |  |  |
| Singing | 3.55 (0.68) | 3.70 (0.74) | 3.34 (0.79) | 3.61 (0.85) |
| Painting, drawing, printmaking or sculpture | 3.68 (0.66) | 3.80 (0.74) | 3.38 (0.80) | 3.88 (0.78) |
| Gardening | 3.55 (0.67) | 3.68 (0.73) | 3.38 (0.76) | 3.55 (0.76) |
| Reading novels, stories, poetry or play | 3.48 (0.69) | 3.83 (0.75) | 3.27 (0.83) | 3.24 (0.87) |
| Playing a musical instrument | 3.50 (0.68) | 3.67 (0.76) | 3.19 (0.80) | 3.63 (0.81) |
| Cookery or baking | 3.33 (0.69) | 3.47 (0.77) | 3.02 (0.78) | 3.52 (0.78) |
| Textile crafts such as embroidery, crocheting or knitting | 3.67 (0.63) | 3.81 (0.70) | 3.36 (0.76) | 3.86 (0.74) |
| Creative writing | 3.66 (0.66) | 3.48 (0.82) | 3.68 (0.80) | 3.89 (0.77) |
| Dancing | 3.66 (0.63) | 3.91 (0.71) | 3.26 (0.78) | 3.80 (0.75) |
| Photography | 3.42 (0.73) | 3.52 (0.83) | 3.11 (0.82) | 3.64 (0.80) |
| Composing music | 3.63 (0.69) | 3.58 (0.83) | 3.43 (0.84) | 3.92 (0.78) |
| Wood crafts such as carving or furniture making | 3.38 (0.73) | 3.49 (0.84) | 3.02 (0.84) | 3.65 (0.81) |
| Creating artworks or animations on a computer | 3.42 (0.73) | 3.51 (0.84) | 3.02 (0.84) | 3.79 (0.83) |
| Pottery, calligraphy or jewellery making | 3.68 (0.64) | 3.84 (0.72) | 3.30 (0.76) | 3.90 (0.76) |
| Rehearsing or performing in a play/drama/opera/musical theatre | 3.82 (0.62) | 3.83 (0.73) | 3.47 (0.78) | 4.21 (0.67) |
| Making films or videos | 3.48 (0.80) | 3.51 (0.87) | 3.15 (0.90) | 3.82 (0.89) |
| Learning or practising magic tricks or circus skills | 3.37 (0.76) | 3.63 (0.89) | 2.92 (0.88) | 3.57 (0.99) |

*Notes: all factors have a possible scoring of 1-5 with higher scores indicating greater use of the strategies categorised within each factor.*
