# Supplementary material for: How do artistic creative activities regulate our emotions? Validation of the Emotion Regulation Strategies for Artistic Creative Activities Scale (ERS-ACA)
Source: PLoS One. 2019 Feb 5;14(2):e0211362. doi: 10.1371/journal.pone.0211362 (PMC6363280; doi:10.1371/journal.pone.0211362)
Supplement: S3 Table — (DOCX) [file pone.0211362.s003.docx]

**S3 Table: Data norms for all factors**

|  | General Factor | Factor 1 (avoidance strategies) | Factor 2  (approach strategies) | Factor 3  (self-development strategies) |
| --- | --- | --- | --- | --- |
| 10% | 2.72 | 2.71 | 2.33 | 2.60 |
| 20% | 3.06 | 3.14 | 2.67 | 3.00 |
| 30% | 3.28 | 3.43 | 3.00 | 3.20 |
| 40% | 3.44 | 3.57 | 3.17 | 3.60 |
| 50% | 3.61 | 3.71 | 3.33 | 3.80 |
| 60% | 3.72 | 4.00 | 3.50 | 4.00 |
| 70% | 3.89 | 4.14 | 3.67 | 4.00 |
| 80% | 4.06 | 4.29 | 4.00 | 4.40 |
| 90% | 4.39 | 4.71 | 4.33 | 4.80 |

*Notes: all factors have a possible scoring of 1-5 with higher scores indicating greater use of the strategies categorised within each factor.*
