# Supplementary material for: How do artistic creative activities regulate our emotions? Validation of the Emotion Regulation Strategies for Artistic Creative Activities Scale (ERS-ACA)
Source: PLoS One. 2019 Feb 5;14(2):e0211362. doi: 10.1371/journal.pone.0211362 (PMC6363280; doi:10.1371/journal.pone.0211362)
Supplement: S1 File — (DOCX) [file pone.0211362.s004.docx]

**S1 File: The ERS-ACA Scale**

**Overview**

The Emotion Regulation Strategies for Artistic Creative Activities scale (ERS-ACA) assesses the types of strategies used to regulate emotions when people engage in artistic creative activities. It calculates an overall ‘general strategy’ and three sub-scales (avoidance strategies, approach strategies and self-development strategies). The instrument is a validated self-completion scale.

**The scale**

People choose to engage in creative activities for a wide range of reasons, including just for the enjoyment of doing the activity. Regardless of ‘why’ you normally do your chosen activity, how much do you agree with the statements below? **When engaging in [name the artistic creative activity]…**

|  | Strong disagree | Disagree | Neither agree nor disagree | Agree | Strongly agree |
| --- | --- | --- | --- | --- | --- |
| 1. …I can block out any unwanted thoughts or feelings | 1 | 2 | 3 | 4 | 5 |
| 2. …I can contemplate what is going on in my life with a clear mind | 1 | 2 | 3 | 4 | 5 |
| 3. …I can shake off any anxieties in my life | 1 | 2 | 3 | 4 | 5 |
| 4. …I feel I am in my own little bubble, away from ordinary worries | 1 | 2 | 3 | 4 | 5 |
| 5. …I feel more confident in myself | 1 | 2 | 3 | 4 | 5 |
| 6. …it boosts my self-esteem | 1 | 2 | 3 | 4 | 5 |
| 7. …it gives me a sense of purpose | 1 | 2 | 3 | 4 | 5 |
| 8. …it helps me forget about my worries | 1 | 2 | 3 | 4 | 5 |
| 9. …it helps me refocus on what matter in my life | 1 | 2 | 3 | 4 | 5 |
| 10. …it helps me to come to terms with my own emotions | 1 | 2 | 3 | 4 | 5 |
| 11. …it helps me to disengage from things that are bothering me | 1 | 2 | 3 | 4 | 5 |
| 12. …it helps me to put worries or problems I have in perspective | 1 | 2 | 3 | 4 | 5 |
| 13. …it helps me to understand my own feelings on things that are on my mind | 1 | 2 | 3 | 4 | 5 |
| 14. …it makes me feel detached from negative things in my life | 1 | 2 | 3 | 4 | 5 |
| 15. …It makes me feel stronger in myself | 1 | 2 | 3 | 4 | 5 |
| 16. …it makes me reflect on my emotions | 1 | 2 | 3 | 4 | 5 |
| 17. …it reaffirms my identity | 1 | 2 | 3 | 4 | 5 |
| 18. …it redirects my attention so I forget unwanted thoughts and feelings | 1 | 2 | 3 | 4 | 5 |

**Scoring**

Scoring takes the average from all items within each factor.

- General factor: sum the total score for all items, then divide by 18
- Factor 1 (avoidance strategies): sum the total score for items 1, 3, 4, 8, 11, 14, 18, then divide by 7
- Factor 2 (approach strategies): sum the total score for items 2, 9, 10, 12, 13, 16, then divide by 6
- Factor 3 (self-development strategies): sum the total score for items 5, 6, 7, 15, 17, then divide by 5
